# Human and Murine Hematopoietic Stem Cell Aging Is Associated with Functional Impairments and Intrinsic Megakaryocytic/Erythroid Bias

**DOI:** 10.1371/journal.pone.0158369

**Published:** 2016-07-01

**Authors:** Alexandra Rundberg Nilsson, Shamit Soneji, Sofia Adolfsson, David Bryder, Cornelis Jan Pronk

**Affiliations:** 1 Medical Faculty, Division of Molecular Hematology, Institution for Laboratory Medicine, Lund University, Lund, Sweden; 2 Medical Faculty, Lund Stem Cell Center, Lund University, Lund, Sweden; 3 Department of Pediatric Oncology/Hematology, Skåne University Hospital, Lund, Sweden; B.C. Cancer Agency, CANADA

## Abstract

Aging within the human hematopoietic system associates with various deficiencies and disease states, including anemia, myeloid neoplasms and reduced adaptive immune responses. Similar phenotypes are observed in mice and have been linked to alterations arising at the hematopoietic stem cell (HSC) level. Such an association is, however, less established in human hematopoiesis and prompted us here to detail characteristics of the most primitive human hematopoietic compartments throughout ontogeny. In addition, we also attempted to interrogate similarities between aging human and murine hematopoiesis. Coupled to the transition from human cord blood (CB) to young and aged bone marrow (BM), we observed a gradual increase in frequency of candidate HSCs. This was accompanied by functional impairments, including decreased lymphoid output and reduced proliferative potential. Downstream of human HSCs, we observed decreasing levels of common lymphoid progenitors (CLPs), and increasing frequencies of megakaryocyte/erythrocyte progenitors (MEPs) with age, which could be linked to changes in lineage-affiliated gene expression patterns in aged human HSCs. These findings were paralleled in mice. Therefore, our data support the notion that age-related changes also in human hematopoiesis involve the HSC pool, with a prominent skewing towards the megakaryocytic/erythroid lineages, and suggests conserved mechanisms underlying aging of the blood cell system.

## Introduction

Processes accompanying aging have attracted increasing research interest in recent years, not least because of a continuously increasing global life expectancy. Concomitantly, the prevalence of age-related diseases increases [1], where elderly individuals often present with alterations in the blood cell system, including reduced adaptive immune responses [2], increased anemia incidence [3, 4], and increased risk of myeloid diseases [5]. Recent insights further suggest that processes underlying physiological aging can resemble pathogenic events in other diseases, such as cancer [6]. Therefore, a deeper understanding of aging processes could benefit not only the aging community, but also younger individuals with diseases reminiscent of aging phenotypes.

Age-related phenotypes within the hematopoietic system can be influenced by cell-extrinsic alterations, such as changes in the bone marrow (BM) microenvironment [7–9]. However, in mice, ample evidence points to intrinsic alterations in the hematopoietic stem cells (HSCs) themselves as the main drivers of hematological aging. These include functional, genetic, and epigenetic modifications [10–16]. In mice, HSCs increase in frequency that however is paralleled by a decreased proliferative capacity on a per-cell basis [10–12, 17]. In several reports, aged murine HSCs have been characterized by an increased myeloid-to-lymphoid output, often referred to as a myeloid bias (My-bi), although also their myeloid cell forming ability is decreased on a per cell basis when compared to younger HSCs [14, 18]. These observations are presumably coupled to an age-related clonal shift within the aged HSC compartment towards increased My-bi HSC frequency at the expense of lymphoid-biased (Ly-bi) HSCs [18–20], although these alterations to some extent can also be strain-specific [18]. Regardless, the lineage skewing with murine HSC aging has been linked to an upregulation of myeloid-specific genes and a downregulation of lymphoid-specific genes [11–15, 18], although many of previous transcriptome analyses were based on a selection and manual curation of lineage-associated genes. By contrast, recent global transcriptome analysis of single HSCs based on more objectively defined lineage-affiliated transcription programs revealed a molecular and functional platelet bias, rather than a My-bi, in aged murine HSC [21].

Human HSC and progenitor cell aging has not been characterized as extensively as within the murine system, but several parallels suggest that aging characteristics at least to some degree might be conserved across species. For instance, HSC proliferation and clonal diversity decline between cord blood (CB) and aged bone marrow (BM) [22–24]. In addition, donor age affects outcome of clinical BM transplantations, although this most likely cannot be solely attributed to reduced HSC performance [25–29]. More direct evaluations of the frequencies and function of aged human hematopoietic stem and progenitor cells (HSPCs) from a limited number of individuals displayed similarities to previous findings in the mouse, including an increased myeloid-to-lymphoid output ratio and decreased reconstitution potential [30], although this is not undisputed [31].

In the present study we characterize age-related changes of human HPSCs and compare these to similar studies in mice. By separating the myeloid lineage into megakaryocytic/erythroid and granulocyte/macrophage lineage, we could reveal a molecular underpinning of megakaryocytic/erythroid bias in aged HSC of both humans and mice. Additionally, we identified a set of genes, commonly and differentially regulated in both aged murine and human HSCs, that hint for species-conserved gene expression patterns of HSC aging.

## Materials and Methods

### Collection of human cord blood and bone marrow cells

Cord blood was received with signed consent from donors at the maternity wards at Lund, Helsingborg and Malmö Hospitals, Sweden. Human bone marrow cells from young donors were obtained with written informed consent from healthy donors between 20 and 26 years of age at Lund Hospital by iliac aspiration. Bone marrow from aged donors, obtained from the orthopedic unit at Hässleholm Hospital, was isolated from waste material of hip replacement surgery (>70 years of age). All procedures were performed with approval from the local ethical committee Regionala Etikprövningsnämnden, Lund (Ethical Approvals LU472/08 and LU Dnr 2009/594) and all research was conducted according to the Declaration of Helsinki. Cord blood samples and bone marrow aspirates from young donors were obtained following written consent. Post hip-surgery waste material to obtain bone marrow samples from aged donors was depersonalized except for donor age and therefor, with approval of the local ethical committee (LU Dnr 2009/594), informed consent was not required.

### Analysis and prospective isolation of human HSC and progenitor cells

Mononuclear cells were isolated using Lymphoprep (Axis-Shield PoC AS, Oslo, Norway) and subsequently CD34-enriched by means of MACS CD34 Microbead Kit (Miltenyi Biotec, Bergish Gladbach, Germany) according to the manufacturers’ instructions. CD34-enriched cells were incubated with Pacific Blue anti-human CD45RA (HI100), FITC anti-human CD34 (4H11), Biotin anti-human CD90 (5E10), PE/Cy7 anti-human CD10 (HI10a), Alexa Fluor 700 anti-human CD45 (HI30), and lineage anti-human PE/Cy5 CD19 (HIB19; initially together with anti-human CD3 [HIT3a], and anti-human CD11b [ICRF44], although later only anti-human CD19 was used as anti-human CD3 and anti-human CD11b did not add any further purity to the CD34 population), all from BioLegend (San Diego, US), PE anti-human CD123 (9F5, BD Biosciences (Franklin Lakes, NJ), and APC anti-human CD110 (BAH-1, BD Pharmingen^™^, Becton Dickinson, Franklin Lakes, NJ). Thereafter, cells were incubated with Brilliant Violet 605-conjugated Streptavidin (BioLegend, San Diego, US) to visualize biotinylated anti-CD90. Propidium Iodide (Molecular Probes) was used to exclude dead cells. Cells were kept cold on ice throughout staining procedures. Cells were acquired on FACSAriaI, FACSAriaIIu, or FACSAriaIII flow cytometers using FACSDiva software (Becton Dickinson, Franklin Lakes, NJ). All flow cytometry data were analyzed with FlowJo software (Tree Star, Ashland, OR).

### Mice

All mice used as bone marrow donors were wild-type C57BL/6 mice. Young murine donors were 10–12 weeks of age and old donors 21–24 months of age. Animals were housed at animal facilities of the Biomedical Center at Lund University and animal experiments were performed with consent from a local ethical committee (Ethical approval LU Dnr M198-06).

### Identification of murine hematopoietic stem and progenitor cells

Murine BM-derived hematopoietic stem and progenitor cells were identified and isolated as previously described [13, 32–34]. In short, BM cells were incubated with a cocktail of antibodies against lineage markers B220, CD4, CD8a, CD11b, Gr-1, Ly6c and Ter119, followed by incubation with antibodies recognizing cKit, Sca1, CD48, CD150, Flt3 and IL7Ra (HSC/CLP stain), or antibodies recognizing cKit, Sca1, CD150, CD41 and CD105 (myeloerythroid stain).

### Functional analysis of human cells

#### Proliferation assay

Freshly isolated, single human HSCs (120 HSCs/donor) were sorted for proliferation analysis into Terasaki plates containing Opti-MEM supplemented with 10% FCS, 2-mercaptoethanol (2-ME), gentamycin, rhSCF (50 ng/ml), hTPO (50 ng/ml), hFLT3-L (50 ng/ml), rhIL-3 (10 ng/ml), EPO (3U/ml), G-CSF (25 ng/ml), IL-6 (25 ng/ml) and IL-11 (15 ng/ml) and kept in a humidified CO_2_ incubator at 37°C for 11 days. Proliferation was scored as: i) wells containing 0–1 cells (no proliferation), ii) wells containing 2–50 cells, and iii) wells containing >50 cells.

#### Assay to assess lymphoid potential

10 human HSCs per well (20–60 wells per donor) were sorted for differentiation analysis in 96-well plates seeded with MS5-cells and medium containing α-MEM supplemented with 10% FCS, 2-ME, gentamycin, rhSCF (10 ng/ml), hFLT3-L (10 ng/ml), rhIL-3 (5 ng/ml), and IL-7 (5 ng/ml). Following seeding, 50% of medium was exchanged with fresh medium containing all cytokines as above, except rhIL-3, once a week. Following 5 weeks of culture, cells were incubated with Al700 anti-human CD45 (HI30), APC anti-human CD11b (ICRF44) and CD33 (WM53), PerCy7 anti-human CD19 (HIB19), all from BioLegend (San Diego, US), as well as APCe780 anti-human CD3 (SK7; eBioscience, San Diego, CA). Propidium Iodine was used to exclude dead cells. Cell analysis was performed on a BD FACS LSRII flow cytometer using FACSDiva software (Becton Dickinson, Franklin Lakes, NJ), and further analyzed using FlowJo software (Treestar, Ashlad, OR).

### Gene expression analysis

Samples containing >10,000 young human HSCs (n = 14), aged HSCs (n = 8), young CLPs (n = 6), young GMPs (n = 6), and young MEPs (n = 6) were sorted into RLT-buffer (QIAGEN, Hilden, Germany) supplemented with 1% 2-ME. Samples were shipped on dry ice to KFB Center of Excellence for Fluorescent Bioanalytics, Regensburg, Germany, prepared and subjected to the Affymetrix Human Gene 1.1 ST array (www.kfb-regensburg.de). Probe level expression values were extracted using RMA algorithm [35] and differentially expressed probes were identified using LIMMA [36]. Probes identified as differential were then hierarchically clustered using the correlation distance measure (1-r) and partitioned by cutting the dendrogram for a specified number of clusters. For subsequent *gene set enrichment analysis (GSEA)* and *high throughout GSEA (BubbleMap)*, population-specific probe sets were narrowed down to only contain one probe for each annotated gene. If more than 500 probes were still present, 500 probes were randomly selected for GSEA. Cluster 1 was chosen as a CLP-specific gene set, cluster 2 as GMP-specific gene set, and cluster 9 as MEP-specific gene set (S1 Fig). When only two populations were compared (young and aged HSCs), the probe showing the highest logarithmic fold change (logFC) was kept for each multiple annotated gene, and the gene set was then ranked over logFC for selection for further analyzes. For young and aged human HSC signatures, probes with an adjusted p-value of < 0.4 were used for GSEA and BubbleMap analyzes to be able to obtain sufficient number of probes for the analyzes. GSEA and BubbleMap analyzes were performed as previously described [37, 38]. Furthermore, differentially expressed genes (DEGs) between young and aged human HSCs with adjusted p-values < 0.1 were analyzed for identification of enriched biological themes using *Gene ontology analysis using Database for Annotation*, *Visualization and Integrated Discovery (DAVID)* as previously described [39]. Microarray data can be accessed at GEO (http://www.ncbi.nlm.nih.gov/geo/) under the accession number GSE69408.

Analyzes of murine HSCs, CLPs, preMegEs, pGM/GMPs, MkPs, and preCFU-Es were done similarly with already published microarray data, accession numbers GSE8407, GSE27686, and GSE44923. Here, cluster 1 was chosen as the CLP-specific gene set, cluster 3 as the pGM/GMP-specific gene set, and cluster 11 as the preMegE-specific gene set in the 3 group comparison with 12 partitions (S2 Fig). For the 4 group comparison with 16 partitions of the murine progenitor populations, cluster 1 was chosen as the CLP-specific gene set, cluster 5 as the MkP-specific gene set, cluster 12 as the pGM/GMP-specific gene set, and cluster 13 as the preCFU-E-specific gene set (S3 Fig). For young and aged murine HSC signatures probes with an adjusted p-value of < 0.05 were used for GSEA and BubbleMap analyzes.

Additionally, the top 500 most significantly expressed DEGs between young and aged HSCs in humans and mice, respectively, were analyzed for conservation using *Ensembl BioMart* [40] and *Microsoft Excel*. Conserved genes were then analyzed for enriched biological themes using *DAVID*.

### Statistics

Functional and frequency results were statistically analyzed and Figures prepared using GraphPad Prism (GraphPad Inc.) software with the indicated statistical formulas.

## Results

### The frequencies of phenotypically defined stem and progenitor cell subsets are altered with age

**Fig 1:**
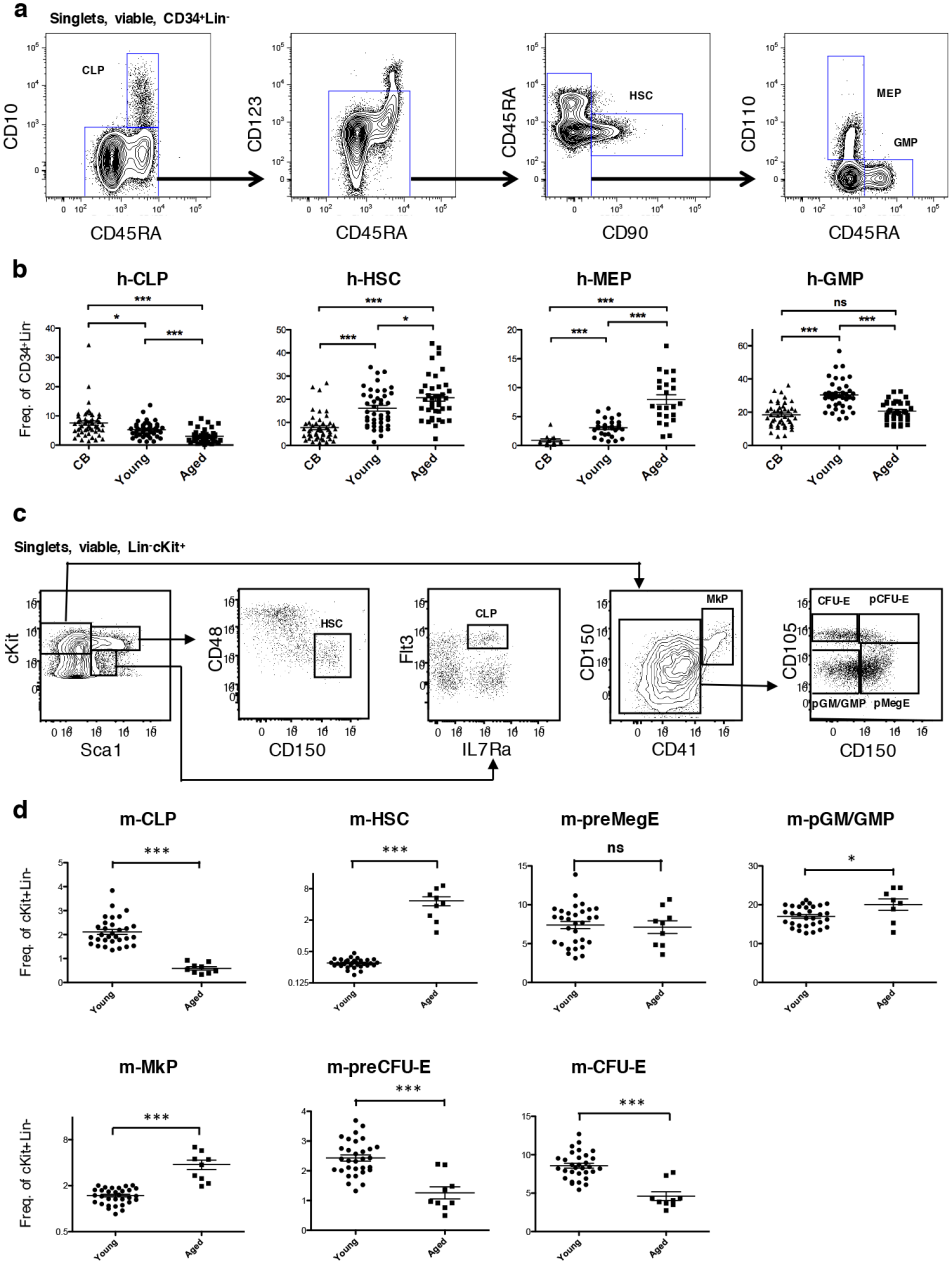
Frequencies of human and murine hematopoietic stem and progenitor cells at different ontogenic stages. **(A-B) Gating strategy and HSPC frequencies for human donors.** **(A)** Flow cytometric profile and gating strategy for h-CLPs (CD10^+^CD45RA^+^), h-HSCs (CD123^low/-^CD45RA^-^CD90^+^), h-MEPs (CD123^low/-^CD45RA^-^CD110^+^) and h-GMPs (CD123^low/-^CD45RA^+^) in one aged bone marrow sample. Pre-gated on viable CD34^+^Lin^-^ singlets. **(B)** Frequencies of depicted early hematopoietic cell types within the primitive CD34^+^Lin^-^ fraction. Each point represents one donor. H-HSCs: aged n = 44, young n = 52, CB n = 58. H-GMPs: aged n = 42, young n = 52, CB n = 45. H-MEPs: aged n = 29, young n = 46, CB n = 13. H-CLPs: aged n = 42, young n = 52, CB n = 49. **(C-D) Gating strategy and HSPC frequencies for mouse donors.** **(C)** Flow cytometric profile and gating strategy for m-HSCs (Sca1^+^Kit^high^CD150^+^CD48^-^), m-CLPs (Sca1^+/-^cKit^low^Flt3^+^IL7Ra^+^), m-MkPs (Sca1-cKit^high^CD150^+^CD41^+^), m-pGM/GMPs (Sca1-cKit^high^CD41^-^CD150^-^CD105^-^), m-pMegEs (Sca1^-^cKit^high^CD41^-^CD150^+^CD105^-^), m-pCFU-Es (Sca1^-^cKit^high^CD41^-^CD150^+^CD105^+^) and CFU-Es (Sca1^-^cKit^high^CD41^-^CD150^-^CD105^+^) in one old murine bone marrow sample. Pre-gated on viable cKit+Lin- singlets. **(D)** Frequencies of depicted early hematopoietic cell types within the primitive Lin^-^cKit^+^ fraction. Each point represents one donor. Young n = 31, aged n = 9. Analyses were performed with unpaired t-tests. * p ≤ 0.05, ** p ≤ 0.01, *** p ≤ 0.001. Reference lines depict means ± SEM.

To evaluate and compare potential aging-related alterations within the primitive human and murine HSPCs, we determined their distribution within the immature CD34^+^Lin^−^ (human) and cKit^+^Lin^−^ (murine) compartments, respectively. For the primitive human HSPCs, we determined the frequencies of the HSC-enriched compartment (CD123^low/−^CD45RA^−^CD90^+^, hereafter referred to as human HSCs, h-HSCs) [41–43], the megakaryocytic/erythroid progenitor-enriched compartment (h-MEPs; CD123^low/−^CD45RA^−^CD110^+^) [44], the common lymphoid progenitor-enriched compartment (h-CLPs; CD45RA^+^CD10^+^) [45], and of the granulocyte/macrophage progenitor-enriched compartment (h-GMPs; CD123^low/−^CD45RA^+^) [46] within the CD34^+^Lin^−^ populations of CB, young BM and aged BM (Fig 1A and 1B). The h-HSC frequency increased from 7.7 ± 0.8% in CB, to 16.1 ± 1.2% in young BM (p < 0.0001) and to 20.6 ± 1.5% in aged BM (p < 0.05). Similarly, the h-MEP frequency was also gradually higher, from 0.9 ± 0.3% in CB, 3.1 ± 0.3% in young BM (p < 0.0001) and 7.9 ± 0.8% in aged BM (p < 0.0001). The h-GMP frequency increased from 18.4 ± 1.1% in CB to 30.4 ± 1.4% in young BM (p < 0.0001), but decreased to 20.7 ± 0.9% in aged BM (p < 0.0001), with no significant difference in aged BM compared to CB. Compared to h-HSCs and h-MEPs, h-CLPs showed an opposite trend, with 7.6% ± 0.8% in CB, decreasing to 5.3% ± 0.4% in young BM (p < 0.05), and further to 3.0% ± 0.4% in aged BM (p < 0.001).

To evaluate if similar alterations could be observed in the murine hematopoietic system, we performed frequency analysis of HSPCs in young and aged mouse BM populations (Fig 1C and 1D). Murine HSCs (m-HSCs) [47] here corresponds to h-HSCs, m-CLPs [48] to h-CLPs, m-preMegE to h-MEPs, and m-pGM/GMP to h-GMPs [32]. Furthermore, to separate the megakaryocytic from the erythroid lineage, megakaryocyte- (m-MkP) and erythrocyte-restricted (m-pCFU-E and m-CFU-E) [32] populations were also analyzed (Fig 1C and 1D). Similar to that observed in the human system, m-HSCs increased in frequency with age (0.3 ± 0.0% in young vs. 4.7 ± 0.9% in aged, p < 0.0001) whereas m-CLPs decreased with age (2.1 ± 0.1% vs. 0.6 ± 0.1%, p < 0.0001; Fig 1D). We observed no significant difference of the m-preMegE population (7.4 ± 0.5% vs. 7.1 ± 0.8%, p < 0.776). However, when exploring the direct progeny of the m-preMegEs, we could detect significantly higher levels of m-MkPs (1.5 ± 0.1% vs. 3.8 ± 0.5%, p < 0.0001), together with significantly lower levels of m-preCFU-Es (2.4 ± 0.1% vs. 1.3 ± 0.2%, p < 0.0001) and m-CFU-Es (8.6 ± 0.3% vs. 4.6 ± 0.6%, p < 0.0001) in the aging BM. The m-pGM/GMP population exhibited a small increase from young to aged BM (17.0 ± 0.5% vs. 20.0 ± 1.5%, p < 0.05). Taken together, these results established alterations in the composition of human and murine HSPC subsets between different ontogenic stages.

### Aging is associated with impaired h-HSC functions

**Fig 2:**
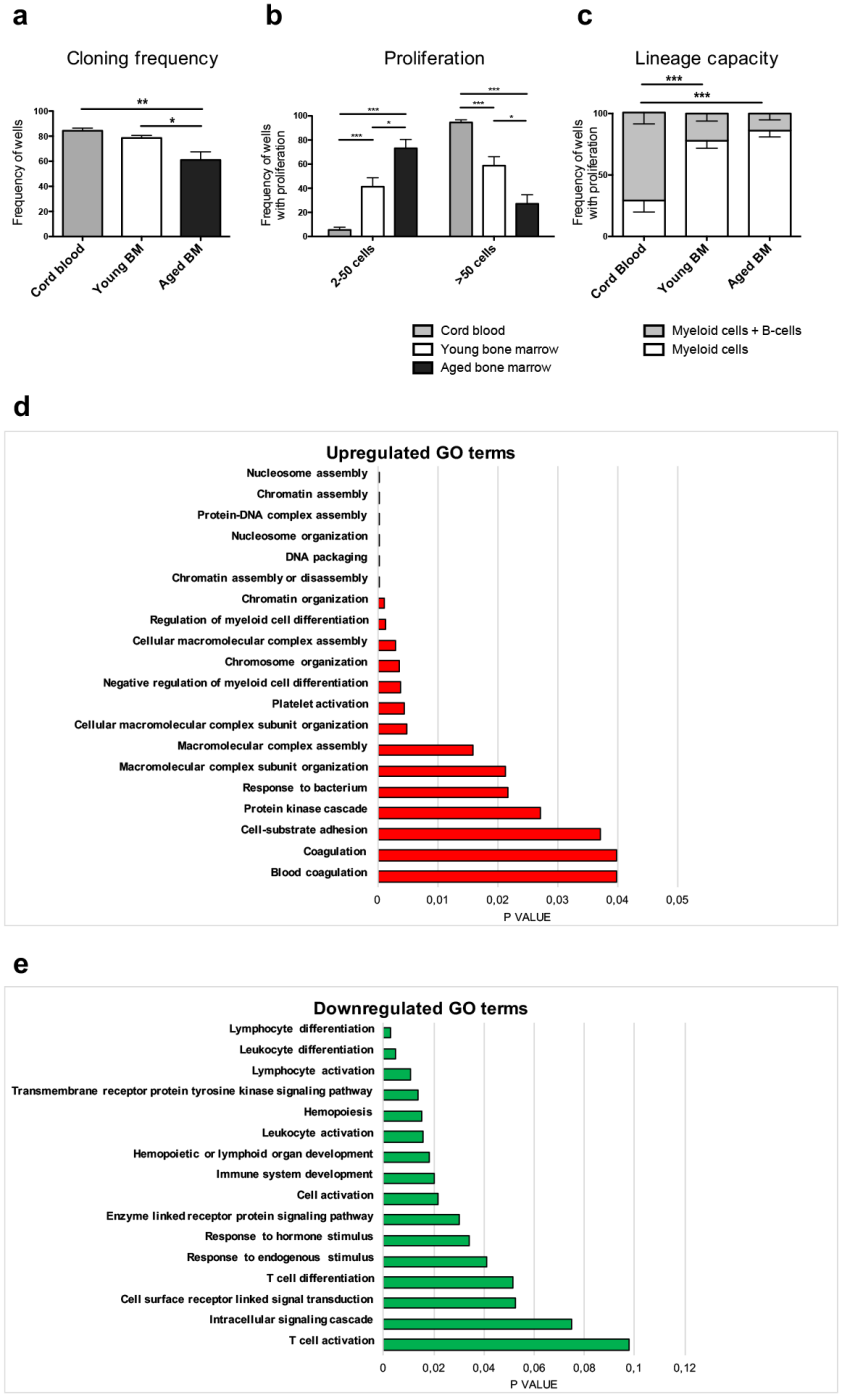
**(A-B) Proliferation of human HSCs at different ontogenic stages.** **(A)** Fractions of candidate h-HSCs (cloning frequency) that proliferate *in vitro*. Cloning frequency was determined as the frequency of wells in which single cells had been sorted and two or more cells was observed after eleven days in culture. **(B)** Clonal size of proliferating h-HSCs, scored as wells containing either 2–50 cells or >50 cells among wells that showed proliferation. Error bars represent +SEM. CB n = 6, young n = 4, aged n = 4. **(C) Lymphoid potential of candidate HSCs.** 10 h-HSCs each were sorted into multiple wells (20–60/donor) and scored after 5 weeks in culture. Graph depicts lineage distribution from wells with proliferation. Error bars represent -SEM. CB n = 6, young n = 10, aged n = 6. Analyses were done with unpaired t-tests. *p ≤ 0.05, **p ≤ 0.01, ***p ≤ 0.001. **(D-E) Up- and downregulated GO terms in aged human BM HSCs.** Differentially expressed genes between young and aged human HSCs with adjusted p-values < 0.1 were subjected to analysis of enriched gene ontology (GO) terms using DAVID. **(D)** Top 20 GO terms of upregulated genes in aged HSCs. **(E)** GO terms of conserved downregulated genes in aged HSCs.

To explore a possible link between the observed alterations of h-HSC frequencies and performance, we next assessed h-HSC function at different ontogenic stages. *In vitro* evaluation of h-HSC cloning frequency (i.e. the proportion of h-HSCs that could be induced to proliferate) revealed decreasing cloning frequencies of 84.3 ± 2.09% in CB to 78.50 ± 2.10% in young BM (p < 0.01), and to 61.00 ± 6.52% in aged BM (p < 0.05; Fig 2A). H-HSC aging was also accompanied by a decreased proliferative capacity (i.e. the clone size of proliferating cells) (Fig 2B).

We next investigated whether age-associated changes in the frequencies of lineage-committed progenitors could be linked to the abilities of h-HSCs to produce myeloid and lymphoid progeny. This was examined via *in vitro* co-culture of h-HSCs with MS5 stroma cells–a system that supports both myeloid and B-lymphoid growth [49]. The frequency of colonies exhibiting lymphoid growth was 71.62 ± 9.28% for CB and was reduced to 22.12 ± 6.12% in young BM (p < 0.0001). A further reduction was observed of HSCs from young BM to 12.87 ± 8.38% in aged BM (Fig 2C), although this failed to reach significance when comparing young and adult BM h-HSCs.

To evaluate whether the altered frequencies and function of aging BM h-HSCs could be more directly linked to any molecular properties, we next performed genome-wide gene expression analysis of prospectively FACS-isolated h-HSCs from 14 young and 8 aged BM donors. Samples were subjected to the Affymetrix Human Gene 1.1 ST array (www.kfb-regensburg.de), and probe level expression values were extracted using RMA algorithm [35]. Differentially expressed probes were identified using LIMMA [36]. Gene ontology analysis of the most differentially expressed genes (DEGs) between young and aged h-HSCs (S1 Table) showed that upregulated genes with h-HSC aging were enriched for biological themes such as chromatin organization, platelet activation, coagulation, and protein kinase cascade (Fig 2D, S2 Table). Aged h-HSCs exhibited downregulation of genes involved in lymphocyte activation and differentiation, immune system development, and cellular pathways, including transmembrane receptor protein tyrosine kinase signaling pathway, response to hormone stimulus, and intracellular signaling pathway (Fig 2E, S2 Table). These results support the notion that at least some of the alterations observed in the aging human BM can be linked to changes originating at the h-HSC level.

### The transcriptional profiles of BM HSCs can be linked to the observed age-related alterations in lineage capacitates

**Fig 3:**
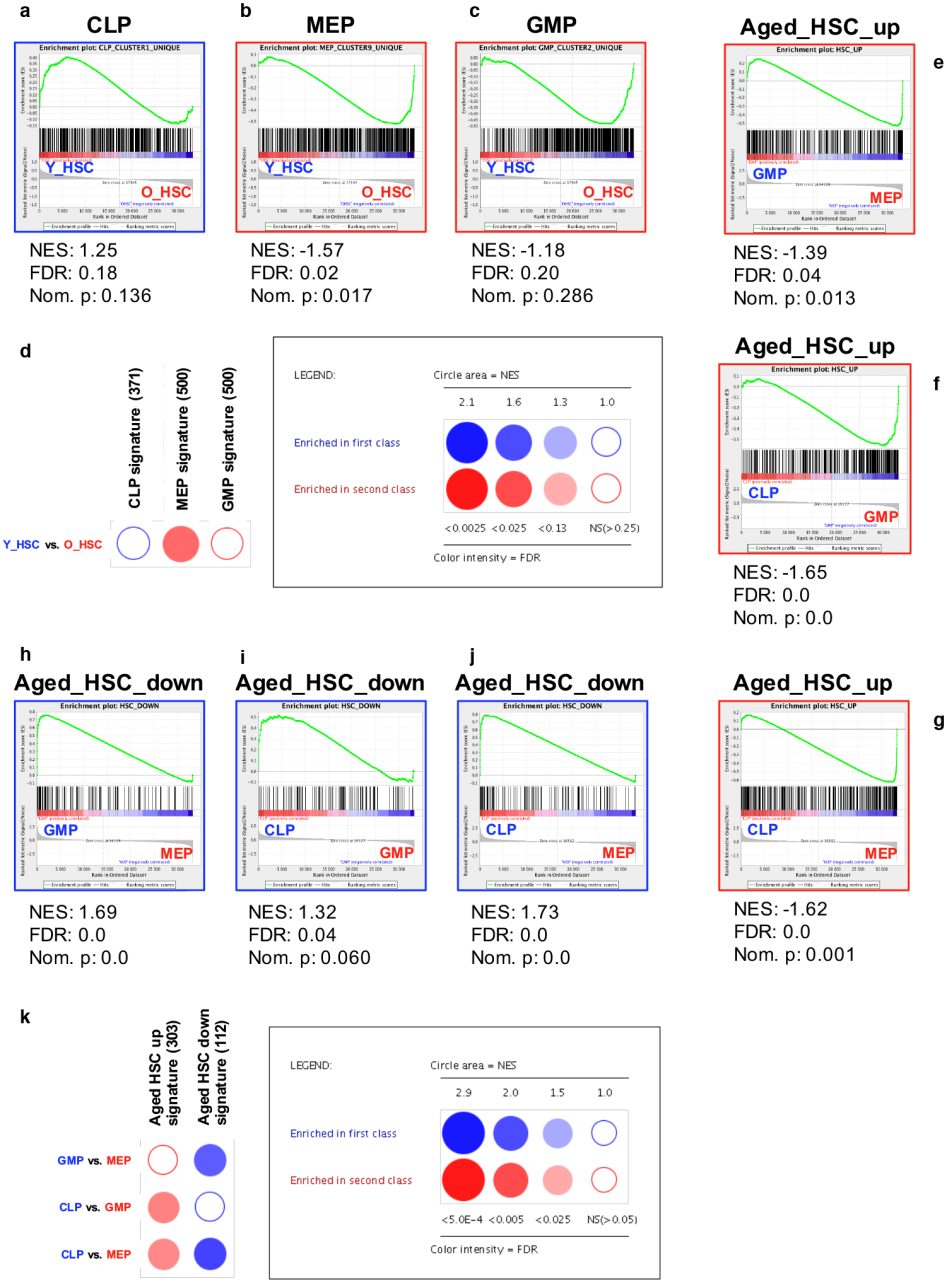
Enrichment of age- and lineage-specific gene expression signatures for human HSPCs. **(A-D): Age-associated enrichment of lineage-affiliated signatures.** Conventional GSEA against lineage-associated gene sets of h-CLPs **(A)**, h-MEPs **(B)** and h-GMPs **(C)** for differential enrichment between young (left) and aged (right) BM h-HSCs (permutation type: phenotypes, FDR < 0.25). Significantly enriched signatures are marked with blue borders if enriched to the left, and with red borders if enriched to the right. **(D)** High throughput GSEA of progenitor-associated signatures to young and aged h-HSCs using BubbleMap. Saturation of bubbles reflects significance (permutation type: phenotypes, B-Y FDR < 0.25). Color represents enrichment to the left side (blue, young) or to the right side (red, aged). The size of the bubbles reflects quantification of the nominal enrichment score (NES). The number of probes in respective gene sets are indicated within brackets. **(E-K): Lineage-associated enrichment of age-affiliated signatures. (E-J)** Conventional GSEA against age-associated h-HSC gene sets for differential enrichment to the depicted lineages (permutation type: gene sets, FDR < 0.05). Significantly enriched signatures are marked with blue borders if enriched to the left, and with red borders if enriched to the right. **(K)** High throughput GSEA of age-associated h-HSC signatures between h-CLPs, h-MEPs, and h-GMPs using BubbleMap. Saturation of bubbles reflects significance (permutation type: gene sets, B-Y FDR < 0.05). Color represents enrichment to the left (blue) or right (red) side.

In parallel with the transcriptional profiling of young and aged h-HSCs, we conducted expression analyses of h-MEPs, h-GMPs, and h-CLPs from 6 young BM donors. We chose this approach to objectively define the transcriptional profiles associated with lineage commitment. To evaluate enrichment of lineage-affiliated signatures (h-MEPs, h-GMPs, and h-CLPs; S3 Fig, S3 Table) to young or aged HSCs, we performed “conventional” gene set enrichment analysis (GSEA) [37] and also more high throughput GSEA BubbleMap analysis, which assesses enrichment between all possible pairwise comparisons [38] (Fig 3A–3D, S4 Table). Using conventional GSEA we found that both h-MEP-associated and h-GMP-associated signatures strongly associated with aged h-HSCs (Fig 3B and 3C). By contrast, the h-CLP-associated signature displayed a significant enrichment to young h-HSCs (Fig 3A). When applying BubbleMap analysis, only h-MEP-associated genes remained significantly enriched to aged h-HSCs (Fig 3D, S4 Table), indicating that the h-MEP-skewing in aged h-HSCs is the most prominent of the three comparisons made.

We also reversed our analyses to evaluate whether up- and downregulated signatures in aging h-HSCs (S3 Table) were particularly enriched in any of the lineage-committed progenitor populations (Fig 3E–3K). Using conventional GSEA, genes specifically upregulated in aged h-HSCs showed a clear enrichment among both h-MEPs and h-GMPs compared to h-CLPs (Fig 3F–3G), with a stronger association to h-MEPs than to h-GMPs (Fig 3E). For the genes downregulated in aged h-HSCs, there was a strong correlation with h-CLPs compared to both h-MEPs and h-GMPs (Fig 3I and 3J), and with h-GMPs when compared to h-MEPs (Fig 3H). BubbleMap analysis showed a similar enrichment of upregulated genes in aged h-HSCs to h-MEPs, but failed to recreate a significant enrichment to h-MEPs compared to h-GMPs (Fig 3K, S4 Table). Furthermore, BubbleMap revealed significant enrichments of downregulated genes to h-CLPs and h-GMPs compared to h-MEPs, but not when h-CLPs were compared to h-GMPs.

**Fig 4:**
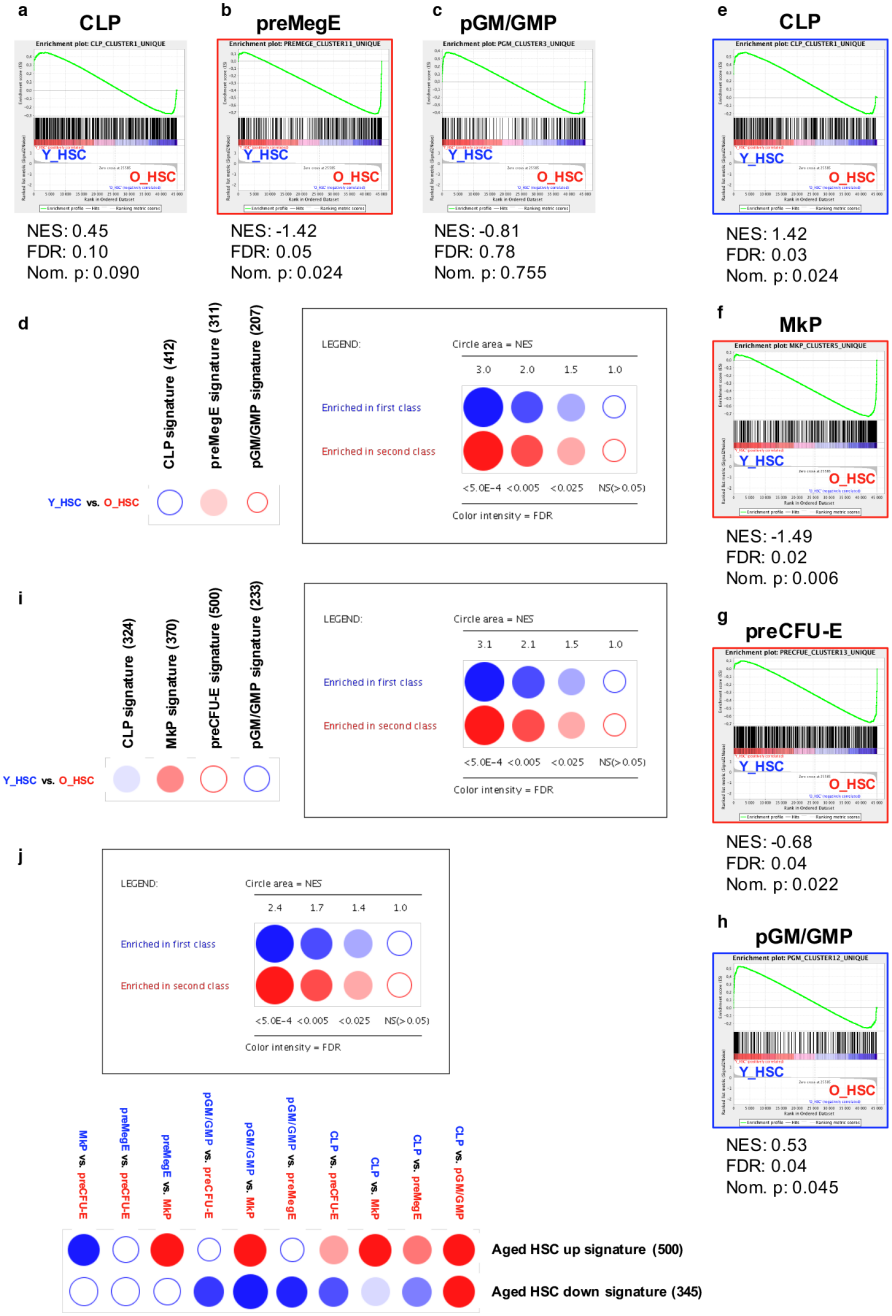
Enrichment of age- and lineage-specific gene expression signatures for mouse HSPCs. **(A-I): Age-associated enrichment of lineage-affiliated signatures.** Conventional GSEA against lineage-associated gene sets generated by comparison of the three progenitor populations m-CLPs **(A)**, m-preMegEs **(B)** and m-pGM/GMPs **(C)** for differential enrichment between young (left) and aged (right) BM m-HSCs. Significantly enriched signatures are marked with blue borders if enriched to the left, and with red borders if enriched to the right (permutation type: gene sets, B-Y FDR < 0.05). **(D)** High throughput GSEA of the progenitor-associated signatures used in A-C to young and aged m-HSCs using BubbleMap (permutation type: gene sets, B-Y FDR < 0.05). **(E-H)** Conventional GSEA against lineage-associated gene sets generated by comparison of the four progenitor populations m-CLPs **(E)**, m-MkPs **(F)**, preCFU-Es **(G)** and m-pGM/GMPs **(H)** for differential enrichment between young (left) and aged (right) BM m-HSCs (permutation type: gene sets, FDR < 0.05). **(I)** High throughput GSEA of the progenitor-associated signatures used in E-H to young and aged m-HSCs using BubbleMap (permutation type: gene sets, B-Y FDR < 0.05). **(J) Lineage-associated enrichment of age-affiliated signatures.** High throughput GSEA of age-associated m-HSC signatures between m-CLPs, m-preMegEs, MkPs, preMegEs, preCFU-Es, and m-GM/GMPs using BubbleMap (permutation type: gene sets, B-Y FDR < 0.05).

To evaluate if a similar lineage-skewing pattern could be reproduced in the murine system, we performed GSEA analyses on murine HSPC subsets (Fig 4, S4 Fig, S5 and S6 Tables). Enrichment analyses of progenitor-specific signatures between m-CLPs, m-preMegEs, and m-pGM/GMPs (S2 Fig, S5 Table) to young and aged m-HSCs revealed a significant enrichment of m-preMegEs to aged m-HSCs both using conventional GSEA (Fig 4B) and BubbleMap (Fig 4D, S6 Table). When instead creating lineage-specific signatures based on differential expression between the four progenitors m-CLPs, m-MkPs, m-preCFU-Es and m-pGM/GMPs (S3 Fig, S5 Table), conventional GSEA revealed significant enrichments of both m-CLP- and m-pGM/GMP-specific signatures to young m-HSCs (Fig 4E and 4H), and of both m-MkP and m-preCFU-E signatures to aged m-HSCs (Fig 4F and 4G). BubbleMap analysis revealed significant enrichments of m-CLPs to young m-HSCs and of m-MkPs to aged m-HSCs (Fig 4I, S6 Table). Moreover, m-preCFU-Es demonstrated a close to significant enrichment to aged m-HSCs as well. When reversing the analysis to evaluate young and aged m-HSC-specific signatures (S5 Table) to progenitors, conventional GSEA demonstrated striking significant enrichments of the upregulated signature in aged m-HSCs to m-MkPs compared to all other evaluated progenitor populations (S4 Fig). The upregulated aged m-HSC signature was also enriched to m-preMegEs, m-pGM/GMPs and m-preCFU-Es compared to m-CLPs. The downregulated signature, on the other hand, enriched strongly with m-pGM/GMPs compared to all other progenitors, and to m-CLPs compared to all other progenitors except for m-pGM/GMPs. Furthermore, the downregulated signature enriched to m-preMegEs compared to both m-MkPs and m-preCFU-Es and to m-MkPs compared to m-preCFU-E. BubbleMap analysis reflected the overall same pattern but failed to reach significance for enrichment of the downregulated signature to m-MkPs and m-preMegEs compared to m-preCFU-Es, and of m-preMegEs compared to m-MkPs (Fig 4J, S6 Table).

Together, these analyses point to transcriptional alterations that may underlie the numerical and functional modifications that occur with HSC aging. Enrichment analyses in both humans and mice revealed that aged HSCs were more distinctly associated with megakaryocytic/erythroid genes, whereas young HSCs were most prominently correlated with CLP-associated genes. Genes specific for granulocyte/macrophage progenitors, however, were distributed among both up- and downregulated genes in aging HSCs.

### Conserved biological themes in aging HSCs of humans and mice are enriched for processes involved in lineage regulation

**Fig 5:**
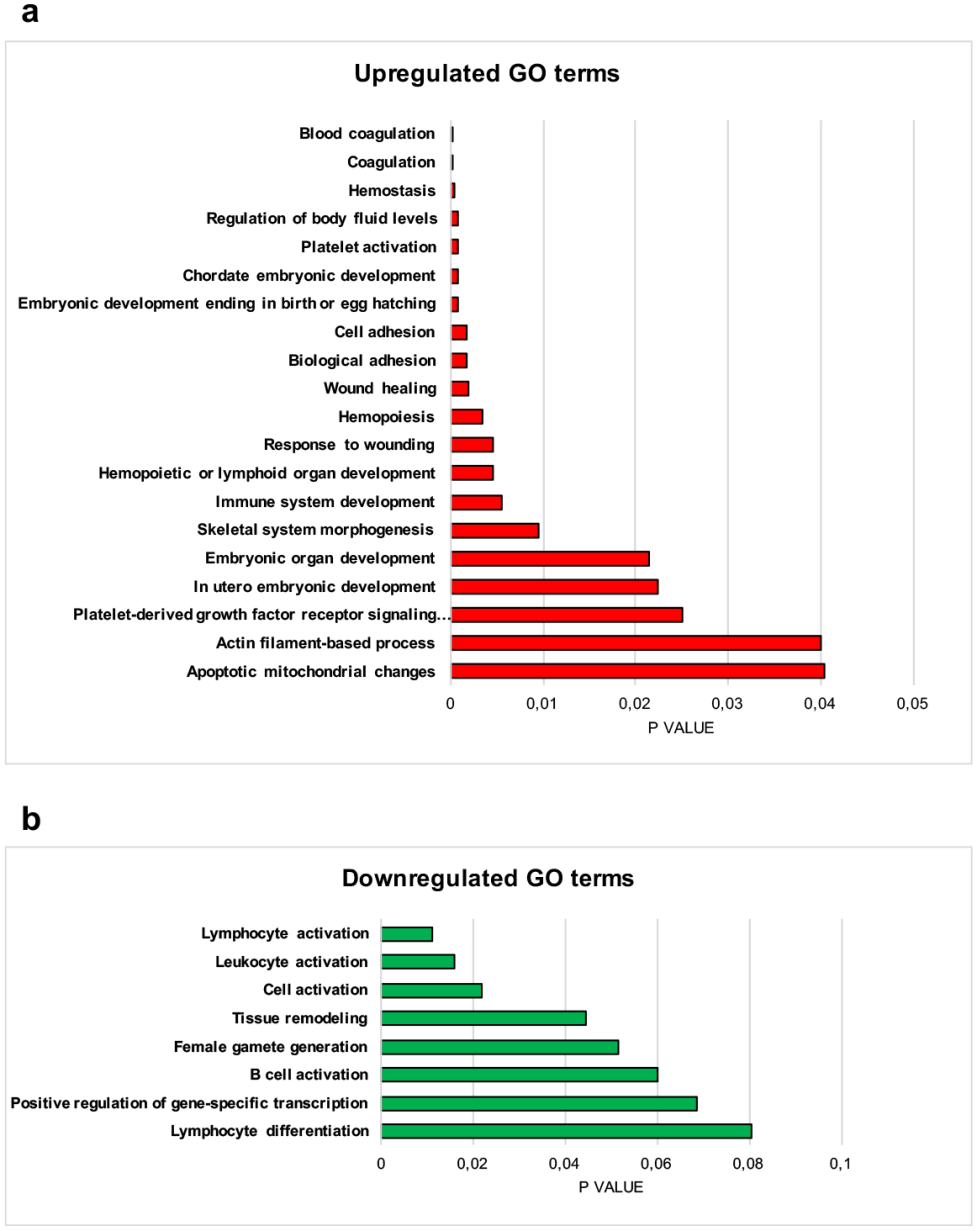
Conserved up- and downregulated GO terms in aging BM HSCs between humans and mice. Conserved genes among the the top 500 most significantly up- and downregulated genes, respectively, in human and mice aged HSCs were subjected to analysis of enriched GO terms using DAVID. **(A)** Top 20 GO terms of conserved upregulated genes in aged HSCs. **(B)** GO terms of conserved downregulated genes in aged HSCs. X-axis indicates p-values of the depicted GO terms.

Finally, to further evaluate the conservation of transcriptional alterations in aging BM HSC populations of humans and mice, we analyzed the enrichment of biological themes among the overlapping genes within the 500 top most significantly DEGs between young and aged HSCs from mice and humans, respectively (S7 and S8 Tables). Conserved upregulated genes (Fig 5A, S8 Table) showed enrichment for megakaryocytic lineage-related GO terms, such as coagulation, platelet activation, and platelet-derived growth factor receptor signaling pathway. Upregulated conserved genes were also enriched for cellular interaction and mobility processes, such as adhesion and actin filament-based process. This list of 24 genes contained several genes that have previously been linked to platelet/erythroid bias in HSCs of humans and/or mice, including *VWF* [21, 50], *ID2* [51], and *ITGB3* [52]. Downregulated conserved genes showed a strong correlation to processes involved in lymphoid activation and differentiation (Fig 5B, S8 Table). This list of 12 genes also included genes that has previously been linked to lymphoid association in mice and/or humans, including *FLT3* [53], *BANK1* [54] and *ITGA4* [55]. These results further support a conserved pattern of upregulated megakaryocytic/erythroid and downregulated lymphoid specification in aging BM HSC compartments between humans and mice.

## Discussion

Continuous growth of the elderly population, paralleled by an increase of age-related diseases, has prompted intense investigations of the mechanisms underlying organismal aging. Insights into these mechanisms could help to prevent or ameliorate age-related phenotypes, as well as improve our understanding and treatments of diseases linked to aging, such as cancer.

In this study, we characterized the aging process within the most primitive compartments of human hematopoiesis, and revealed prominent alterations in between CB to adult and advanced aged BM (Figs 1 and 2). For comparison, we conducted similar analyses in aging murine BM, hypothesizing that conserved themes of human and murine hematopoietic aging might be of evolutional importance and because of the seemingly conflicting data that have been reported in both human [30, 31] and murine [11–15, 18, 21] HSPC aging.

In line with previous findings in the murine system [12, 14, 21, 56] and the results of one previous human study [30], we demonstrate that h-HSC and m-HSC frequencies increases with age (Fig 1B and 1D). By contrast, a previous study on human cells reported more stable h-HSC frequencies with age [31]. This discrepancy may be explained by the relatively small number of biological replicates included in the previous work, together with large biological variations, and/or differences in the candidate HSC population reported on. The latter might be of particular importance as the different ontogenic stages are derived from different organ sources. Thus, reporting the frequency within total MNCs could be misleading, as the samples are likely to contain different amounts of (non-BM) MNCs. Although limited in sample size, stable frequencies of CD34 positive cells within BM cells have previously been reported from comparisons of BM aspirates from young and aged individuals [57], which prompted us to here determine h-HSPC frequencies within the CD34^+^Lin^-^ population. To allow for comparison of human and murine results, we evaluated frequencies of m-HSPCs within the cKit^+^Lin^-^ population, a population that likely is not identical to, but reminiscent of the human CD34^+^Lin^-^ population.

In agreement with previous reports, we also observed decreased CLP frequencies with age in both humans and mice. However, our observations diverge for the frequencies of granulocyte/macrophage and megakaryocyte/erythrocyte progenitors. We, and others [30] did not observe the increase of h-GMPs with age that has been reported in mice [12]. In aged BM, we observed a prominent increase of h-MEP frequency (Fig 1B), but failed to reproduce a similar increase of m-preMegEs in the murine setting (Fig 1D). This discrepancy could potentially be explained by the possibility that the h-MEP and m-preMegE populations are not fully comparable, particularly as the phenotypical identity and the erythroid versus megakaryocytic potentials at different stages of the human hematopoietic development is debated [44, 58, 59]. Moreover, changes in levels of platelets and erythrocytes differ in aged humans and mice, with humans showing decreased levels of both red blood cells (RBCs) [60] and platelets [49, 61], whereas mice exhibits decreased levels of RBCs [62] but increased levels of platelets [21]. When evaluating populations downstream of m-preMegEs in the aging murine BM we could, in agreement with previous observations [21], observe initiation of this pattern with m-MkPs showing an increased frequency, and erythroid progenitors pre-CFU-Es and CFU-Es showing decreased frequencies (Fig 1D).

Age-related increases of HSC frequencies have repeatedly been reported to coincide with cell-intrinsic functional impairments [10–12, 14, 17, 30, 56]. Our present *in vitro* single-cell h-HSC experiments (Fig 2A and 2B), demonstrated that h-HSCs produced continuously smaller colonies from CB to young and aged h-HSCs on a per-cell basis, strongly suggesting that also the human system shows cell-intrinsic impairments of h-HSC proliferation. While it cannot be excluded that cell-extrinsic factors may also impact the age-related decline of human HSC function, it appears that cell-intrinsic factors and the transcriptional responses that accompany these are important contributors to aging HSC phenotypes. We also evaluated lymphoid capacity of h-HSCs from different ontogenic stages by culturing h-HSCs on MS5 stroma layers (Fig 2C). These experiments demonstrated a decreased lymphoid potential of candidate HSCs obtained from BM when compared to CB. While we observed a trend towards further reduction in lymphoid capacity with advanced BM h-HSC aging, this failed to reach significance, although *in vitro* comparisons of lymphoid potential between young and aged BM h-HSCs previously was reported to be significantly reduced with age [30]. Regardless, our data support functional impairments with regards to proliferation and lymphoid capacity in aging HSCs of both humans and mice.

To investigate potential age-related alterations in the lineage-affiliation of HSCs, we designed an experimental approach that allowed objective and unbiased evaluation on gene expression level. Our approach was based on transcriptional profiling of prospectively isolated human cell populations that are enriched for granulocyte/macrophage (h-GMPs), megakaryocytic/erythroid (h-MEPs), and lymphoid (h-CLPs) potential, respectively, to define gene expression signatures associated with early lineage commitment (S1 Fig, S3 Table). In a similar manner, m-CLPs, m-pGM/GMPs, m-pMegEs, m-MkPs and m-pCFU-Es were used to define murine differentially expressed lineage-affiliated gene signatures using two different comparisons–either differential expression between m-CLPs, m-pGM/GMPs, and m-preMegEs (S2 Fig, S5 Table), or between m-CLPs, mpGM/GMPs, m-MkPs, and m-preCFU-Es (S3 Fig, S5 Table). While skewing towards the myeloid lineage has been indicated previously [12–15] in both mice and humans [30], the manual curation of lineage-affiliated genes in previous work has failed to separate between individual myeloid lineages. Our present strategy used h-GMPs as reference for myeloid specification, thereby separating the granulocyte/macrophage lineage (referred to here as the myeloid lineage) from the megakaryocytic/erythroid lineage. Strikingly, and somewhat in contrast to previous findings, our results did not confirm a strong myeloid (granulocyte/monocyte) skewing in aged m-HSCs (Fig 4C, 4D, 4H and 4I). By contrast, aged HSCs showed a clear association with megakaryocyte/erythrocyte-affiliated genes in both humans and mice (Figs 3B, 3D, 4B and 4D). Some of the human h-MEP-associated genes identified in our gene analysis were linked to a common myelo-erythroid lineage used in previous studies [30], including *MICAL3*, *PTGS1*, and *CSF2RB*, which could partly explain the previously reported “myeloid” skewing in humans. Subsequent analysis of the murine populations, separating the bipotent m-pMegE signature into megakaryocytic (m-MkP) and erythroid (m-preCFU-E) signatures, displayed an association of aged m-HSC with both megakaryocytic signature (Fig 4F) and with erythroid signature (Fig 4G). Finally, and in agreement with previous findings in humans [30] and mice [5, 7, 18, 19, 21], we observed a distinct age-associated downregulation of lymphoid-affiliated genes in h- and m-HSCs (Figs 3A, 3D, 4E and 4I), decreased frequencies of phenotypic h- and m-CLPs (Fig 1B and 1D), as well as decreased lymphoid output from h-HSC in vitro (Fig 2C).

It should be noted that the human and murine HSPCs analyzed in this study were isolated solely based on the expression patterns of cell surface markers. Numeral claims in published literature point to an enrichment of cellular characteristics, such as stem cell activity and/or lineage potentials, based on such phenotypic signatures. Still, it is not unlikely that the studied cell populations in our study present with functional impurities that could potentially skew the interpretation of the results. For instance, murine HSCs are isolated to high purity with the phenotypical markers used in this study [47], whereas the human HSC compartment herein appears less enriched for true HSC activity. Also, expression of cell surface markers of any given functional unit could potentially change across ontogeny which was not specifically addressed here. However, many observations in elderly human cells were paralleled by similar observations in aged murine cells. Therefore, we argue for overlapping cellular qualities of phenotypic human and murine HSPCs, as well as overlapping mechanisms that underlie HSC aging. Alterations in the composition of heterogeneous HSC clones that has been observed in aged mice [63] probably contributes to the observed changes also in elderly humans, although this was not addressed herein as single cells were not investigated.

**Fig 6:**
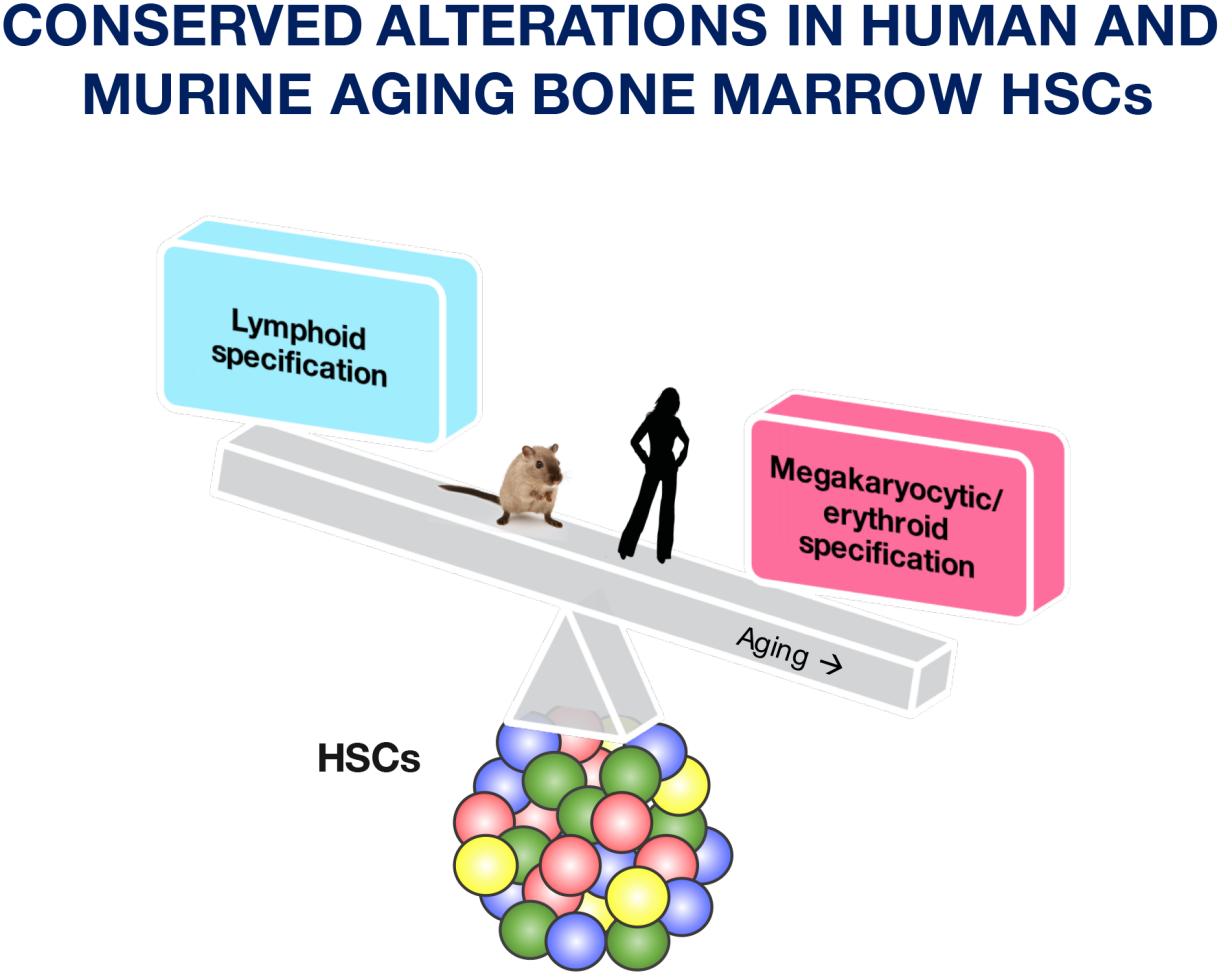
Summary of conserved concepts in aged BM HSCs between humans and mice. Aging of the HSC-enriched compartment of the bone marrow is associated with a decreased lymphoid specification, whereas megakaryocytic/erythroid specification is increased.

In conclusion, we here report intrinsic phenotypic, functional, and transcriptional changes of human and murine HSCs with progressing age (Fig 6). Our results show a megakaryocyte/erythrocyte transcriptional profile that strongly associates with aged HSCs and support the notion that an increased HSC frequency with age may be a compensatory mechanism to sustain sufficient blood cell replenishment. However, these compensatory mechanisms do not fully maintain optimal functions of HSCs and progenitor cells in elderly human, as evidenced by the frequency of age-related hematological defects, including anemia and reduced immune responses [2–4]. A deeper understanding of the events underlying this functional decline may support interventional approaches to prevent or ameliorate the aging hematopoietic phenotype.

## Supporting Information

S1 Fig Population clusters with 12 partitions based on 3 group comparison with the human progenitors CLPs, MEPs, and GMPs. Probe level expression values from Affymetrix data were extracted using RMA algorithm and differentially expressed probes were identified using LIMMA. Probes identified as differential were then hierarchically clustered using the correlation distance measure (1-r) and partitioned by cutting the dendrogram for 12 clusters. Cluster 1 was chosen as a CLP-specific gene set, cluster 2 as GMP-specific gene set, and cluster 9 as MEP-specific gene set.
(TIF)

S2 Fig Populations clusters with 12 partitions based on 3 group comparison with the murine progenitors CLPs, preMegEs, and pGM/GMPs. Cluster 1 was chosen as the CLP-specific gene set, cluster 3 as the pGM/GMP-specific gene set, and cluster 11 as the preMegE-specific gene set.
(TIF)

S3 Fig Populations clusters with 16 partitions based on 4 group comparison with the murine progenitors CLPs, MkPs, preCFU-Es, and pGM/GMPs. Cluster 1 was chosen as the CLP-specific gene set, cluster 5 as the MkP-specific gene set, cluster 12 as the pGM-specific gene set, and cluster 13 as the preCFU-E-specific gene set.
(TIF)

S4 Fig Lineage-associated enrichment of age-affiliated signatures. Conventional GSEA against age-associated m-HSC gene sets for differential enrichment to the depicted lineages (permutation type: gene sets, FDR < 0.05). Significantly enriched signatures are marked with blue borders if enriched to the left, and with red borders if enriched to the right.
(TIF)

S1 Table Up- and downregulated genes for human GO analysis. DEGs between young and aged human HSCs with adjusted p-values < 0.1 that were used for analysis of enriched biological themes using *DAVID*.
(XLSX)

S2 Table Enriched GO terms for genes specifically up- or downregulated in aged human HSCs. Enriched GO terms obtained from DAVID analysis of up- and dowregulated genes in aged human BM HSCs.
(XLSX)

S3 Table Human gene sets. Human gene sets of probes used for gene set enrichment analyses.
(XLSX)

S4 Table Summary of BubbleMap analyzes on human populations. NES, GSEA, and B-Y FDR values obtained during BubbleMap analyses of human gene sets.
(XLSX)

S5 Table Murine gene sets. Mouse gene sets of probes used for gene set enrichment analyses.
(XLSX)

S6 Table Summary of BubbleMap analyzes on murine populations. NES, GSEA, and B-Y FDR values obtained during BubbleMap analyses of mouse gene sets.
(XLSX)

S7 Table Up- and downregulated genes for conserved GO analysis. Conserved genes between humans and mice among the top 500 most significant DEGs between young and aged HSCs for each species used for GO analysis. Significance was scored based on adjusted p values.
(XLSX)

S8 Table Enriched GO terms for conserved genes specifically up- or downregulated in aged human and murine HSCs. Enriched GO terms obtained from DAVID analysis of conserved up- and downregulated genes in aged human and murine BM HSCs.
(XLSX)
