# Supplementary figures and images for: Human and Murine Hematopoietic Stem Cell Aging Is Associated with Functional Impairments and Intrinsic Megakaryocytic/Erythroid Bias
Source: PLoS One. 2016 Jul 1;11(7):e0158369. doi: 10.1371/journal.pone.0158369 (PMC4930192; doi:10.1371/journal.pone.0158369)

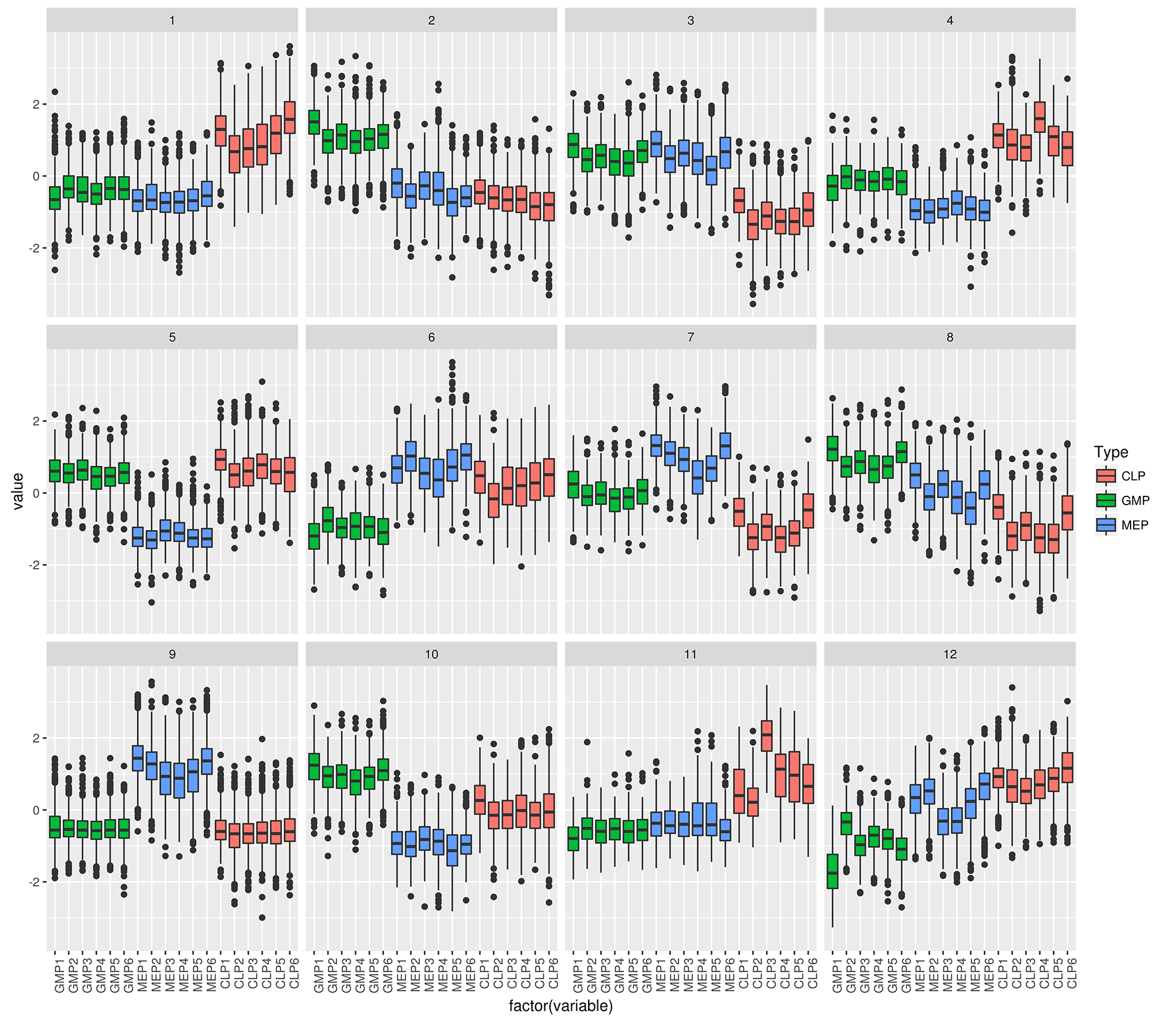

Supplement: S1 Fig — Probe level expression values from Affymetrix data were extracted using RMA algorithm and differentially expressed probes were identified using LIMMA. Probes identified as differential were then hierarchically clustered using the correlation distance measure (1-r) and partitioned by cutting the dendrogram for 12 clusters. Cluster 1 was chosen as a CLP-specific gene set, cluster 2 as GMP-specific gene set, and cluster 9 as MEP-specific gene set. (TIF) [file pone.0158369.s001.tif]

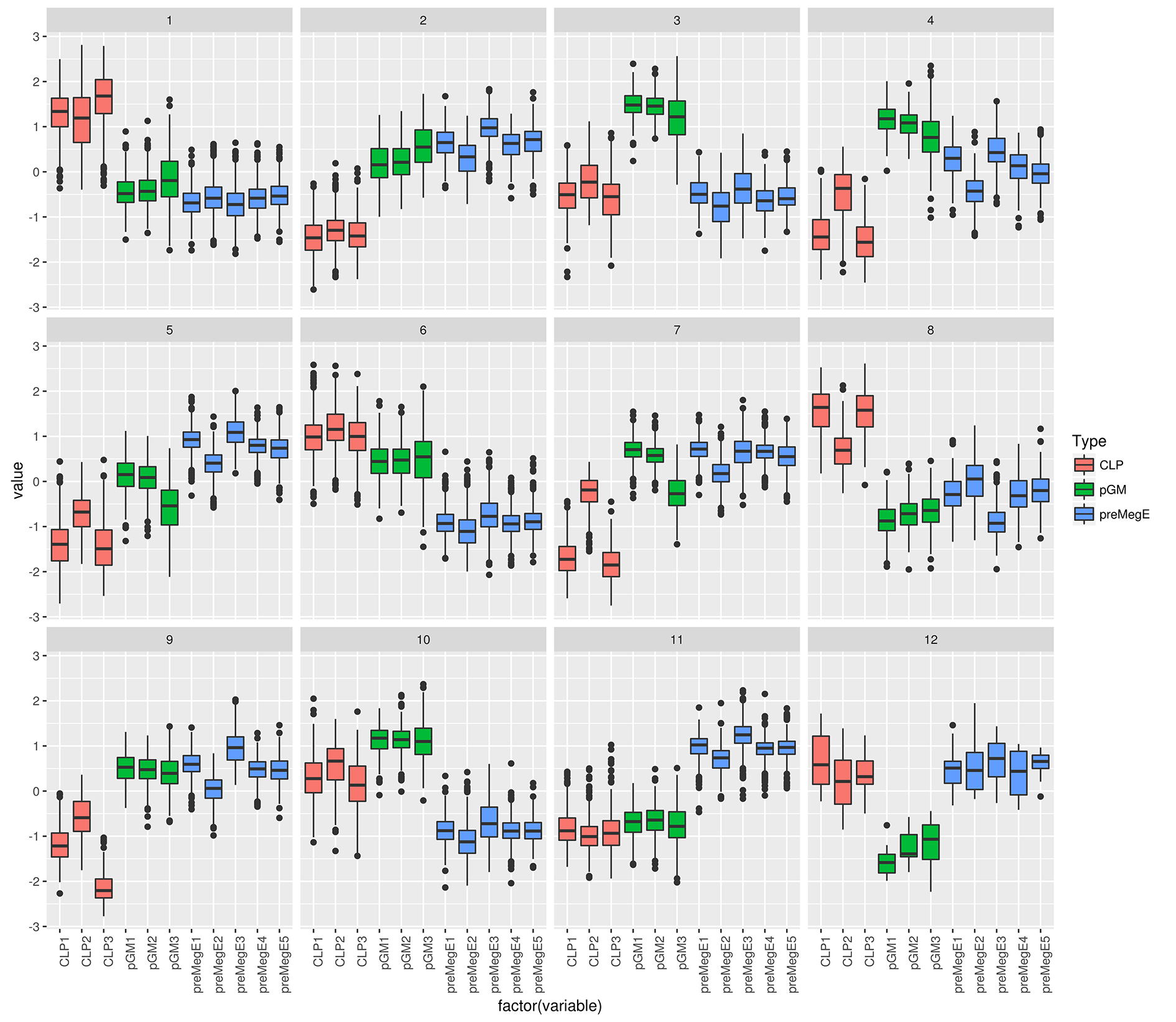

Supplement: S2 Fig — Cluster 1 was chosen as the CLP-specific gene set, cluster 3 as the pGM/GMP-specific gene set, and cluster 11 as the preMegE-specific gene set. (TIF) [file pone.0158369.s002.tif]

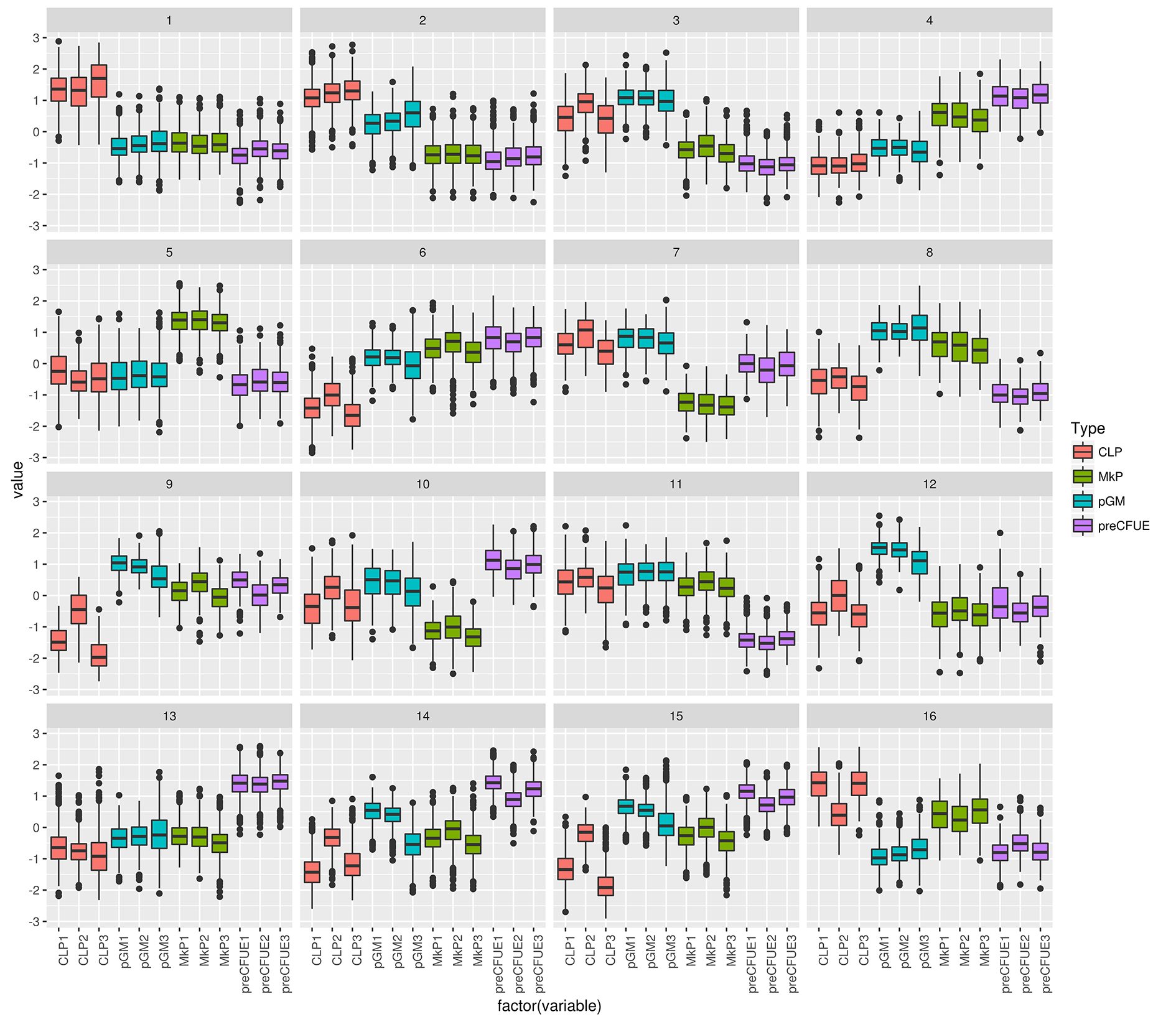

Supplement: S3 Fig — Cluster 1 was chosen as the CLP-specific gene set, cluster 5 as the MkP-specific gene set, cluster 12 as the pGM-specific gene set, and cluster 13 as the preCFU-E-specific gene set. (TIF) [file pone.0158369.s003.tif]

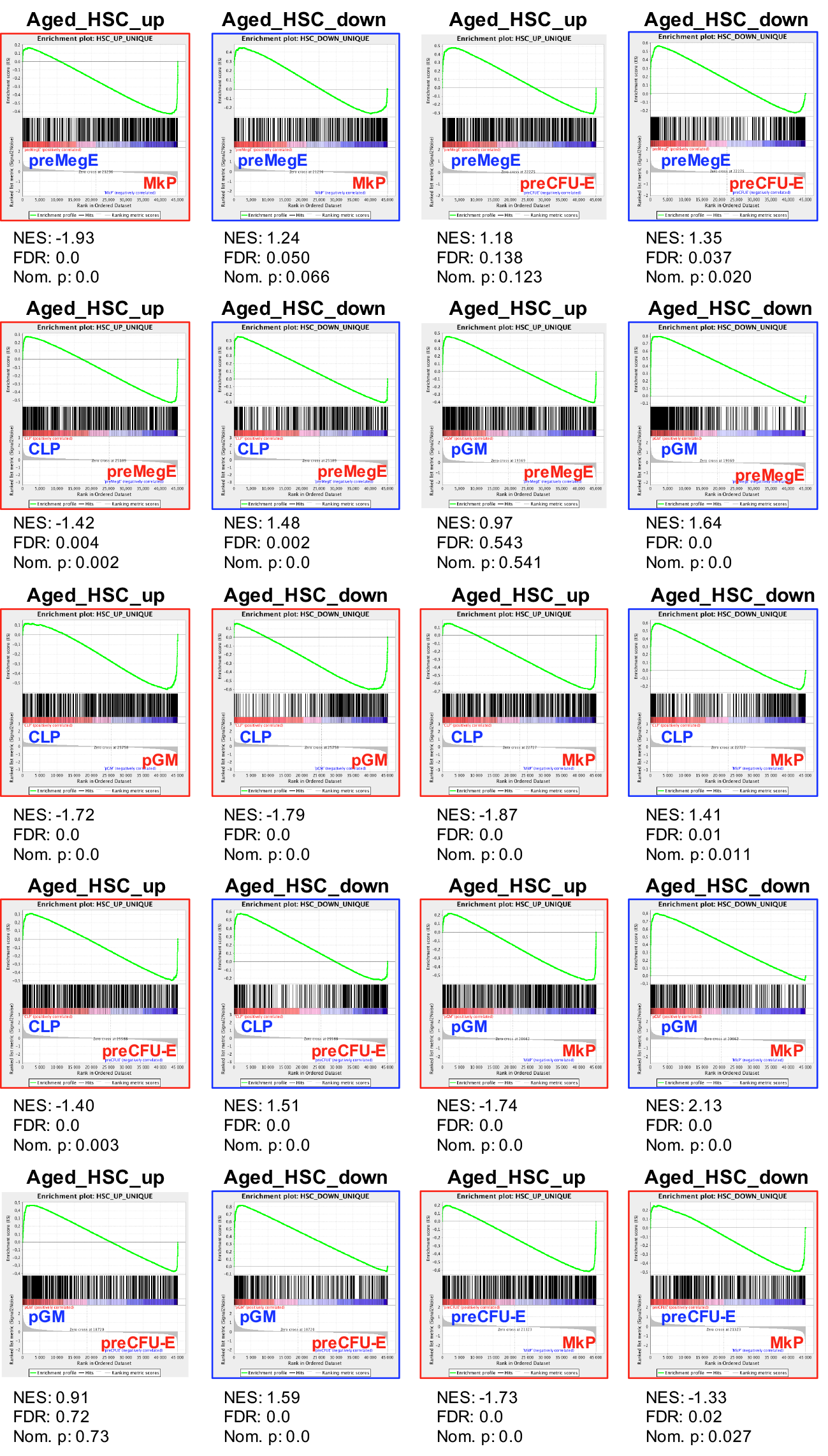

Supplement: S4 Fig — Conventional GSEA against age-associated m-HSC gene sets for differential enrichment to the depicted lineages (permutation type: gene sets, FDR < 0.05). Significantly enriched signatures are marked with blue borders if enriched to the left, and with red borders if enriched to the right. (TIF) [file pone.0158369.s004.tif]
